# Topological structures, spontaneous symmetry breaking and energy spectra in dipole hexagonal lattices

**DOI:** 10.1038/s41598-021-83359-x

**Published:** 2021-02-18

**Authors:** Josep Batle

**Affiliations:** Departament de Física, Universitat de les Illes Balears, 07122 Palma de Mallorca, Balearic Islands Spain

**Keywords:** Magnetic properties and materials, Structure of solids and liquids

## Abstract

The interplay between the special triangular/hexagonal two dimensional lattice and the long range dipole–dipole interaction gives rise to topological defects, specifically the vortex, formed by a particular arrangement of the interacting classic dipoles. The nature of such vortices has been traditionally explained on the basis of numerical evidence. Here we propose the emerging formation of vortices as the natural minimum energy configuration of interacting (in-plane) two-dimensional dipoles based on the mechanism of spontaneous symmetry breaking. As opposed to the quantal case, where spin textures such as skyrmions or bimerons occur due to non-linearities in their Hamiltonian, it is still possible to witness classic topological structures due only to the nature of the dipole–dipole force. We shall present other (new) topological structures for the in-plane honeycomb lattice, as well as for two-dimensional out-of-plane dipoles. These structures will prove to be essential in the minimum energy configurations for three-dimensional simple hexagonal and hexagonal-closed-packed structures, whose energies in the bulk are obtained for the first time.

## Introduction

Considerable progress has occurred in recent years both experimentally and theoretically with regards to the description of dipolar systems with large dipole moments^1–3^. In the low-temperature regime, it is plausible to consider the formation of classical crystals of dipolar particles. Systems of dipoles whose orientation is perpendicular to the plane of motion have been considered in the past, either classically^4–7^ or quantum mechanically^8–13^.

Depending on several quantum effects, dipolar interactions can describe unexpected properties in helium^14^, as well as accounting for particular effects observed in magnetic colloids^15,16^. It is well-known that in most realistic cases, the dipole–dipole interaction is weak compared to other interactions or thermal fluctuations. Its strength is usually measured by the parameter $\lambda \,=\,\frac{\mu _0 \mu ^2}{4\pi k_B T a^3}$, with *a* being the lattice constant and $\mu$ the magnetic dipole moment. Due to the typical length scales in ferrofluids at room temperatures, the value of $\lambda$ never exceeds 5. Thus, we have assumed throughout the present contribution that we can work in the regime of strong dipole moments, which in turn are responsible for the structural stability and ordering of matter.

Luttinger and Tisza^17^ treated in their seminal paper the formation of crystals composed solely by dipoles. In their work, they carried out an elegant method for finding the entire energy per dipole spectra in the thermodynamic limit for several three-dimensional structures. This work paved the way for numerical and analytical studies of other systems that were amenable to a classical treatment, either in two or three dimensions.

In recent years, the numerous improvements of surface technology in ultra thin films have unveiled several magnetic patterns with different symmetries as a function of temperature and lattice structure^18–20^. The magnetization distribution within such patterns describe topological defects such as vortices, domains, and walls^18–20^. Although real materials have to be addressed quantum mechanically, the previous topological effects can also be explained in classic terms by means of the long range dipole–dipole interaction.

Besides spintronics, topological matter is an aspiring research field concerned with non-collinear spin textures. The most prominent example is the magnetic skyrmion^21^, a whirl-like nano-object. Its topological protection gives it an enormous stability even at small sizes, which makes it a potential carrier of information in future data storage devices^22–24^. These topologically non-trivial spin textures appear in the quantum realm.

The exploration of topological patterns, specifically vortices, in classic systems is carried out in the present work. The existence of spin textures in quantum systems appeals to the exploration in full detail of the existence of their classical counterparts, based solely on dipole–dipole interactions. For these structures do not seem to occur in any other lattice but the triangular/hexagonal one, we shall study this lattice extensively. The present contribution is divided as follows: in “Motivation”, we provide the tools that motivate our study. “Luttinger–Tisza and energy decomposion methods” deals with the basics of the Luttinger–Tisza method, alongside our method for finding minimum energies per dipole in arbitrary lattices and dimensions in the thermodynamic limit. The next section studies the energetics and topologies of hexagonal out-of-plane and in-plane dipoles. We invoke the notion of spontaneous symmetry breaking in order to explain the appearance of the vortex structure as the ground state of the in-plane hexagonal instance. Also, the configuration of dipoles and their concomitant minimum energy per particle for the honeycomb lattice is studied in full detail “Two‑dimensional structures”. “Three-dimensional structures” is devoted to obtaining the minimum energy configurations and the corresponding energies for the three-dimensional cases of simple hexagonal and closed-packed lattices, even as function of the interlayer distance. Finally, some conclusions are drawn in “Discussion”.

## Motivation

Two classic identical dipoles of magnitude of $\mu$, $\vec{m}_u$ and $\vec{m}_v$ localized, respectively, at positions $\vec{r}_u$ and $\vec{r}_{v}$, with dipole moments given as

1 $$\begin{aligned} \vec{m} = \mu \begin{pmatrix} \sin (\theta ) \cos (\phi )\\ \sin (\theta ) \sin (\phi ) \\ \cos (\theta ) \end{pmatrix}, \end{aligned}$$

interact with each other giving rise to the interaction energy

2 $$\begin{aligned} E_{u,v} = \mathcal {C}_E \bigg ( \frac{\vec{m}_u\cdot \vec{m}_v}{r_{uv}^3}- 3\frac{ \left( \vec{m}_u\cdot \vec{r}_{uv}\right) \left( \vec{m}_v\cdot \vec{r}_{uv}\right) }{r_{uv}^5} \bigg ), \end{aligned}$$

where $\vec{r}_{uv}$ is the vector between the two dipoles *u* and *v*, $r_{uv}=||\mathbf {r}_{uv}||$ is their separation distance (constant $\mathcal {C}_E$ is either $\frac{\mu _0}{4\pi }$ or $\frac{1}{4\pi \epsilon _0}$, magnetic or electric, respectively). Energy (2) will be given in units of $\mathcal {C}_E\mu ^2/a^3$ throughout the present work.

The terms in (2) possess a well-defined physical meaning. On the one hand, the first term is essentially the classical counterpart of the Heisenberg exchange interaction, which plays a paramount role in magnetism. On the other hand, the second term favors the alignment between the two dipoles. In a particular configuration of dipoles in equilibrium, there is a non-trivial interplay between the two contributions, which, depending on the particular lattice, does not resemble the usual Heisenberg interaction. Furthermore, the Heisenberg Hamiltonian is computed for nearest-neighbors. What will make our study specially challenging is that in the case of interaction (2), one will consider *all* pairs, as opposed to just neighbors as in the usual Heisenberg case, and the coupling *constant* will not be a constant at all, but shall possess a long-range nature (going as $1/r^3$).

Emerging fields of research such as chiral magnetism and topological spintronics are developed through the study of topologically nontrivial spin textures. These structures are found in a variety of magnetic materials^25–34^, and are potentially interesting for information processing and data storage. As opposed to the classic interaction energy functional form (2), the Hamiltonian for quantum spins, running over nearest-neighbor, next-nearest-neighbor, and next-next-nearest-neighbor sites, contains additional non-linear terms that are responsible for the appearance of magnetic skyrmions in 3D or magnetic bimerons in 2D^35^. Also, these topological magnetized spin textures carry integer topological charges *Q*. In essence, it is the existence of non-linearities in the quantum Hamiltonian that lead to well-defined topological structures.

In spite of the stark contrast existing between the nature and form of the interactions in quantum and classical systems, one may wonder if it is still possible to observe special topological structures in classic systems of interacting dipoles. The answer is affirmative in systems with hexagonal symmetry, either in two or three dimensions. To be more specific, we shall focus on the minimum energy configuration of systems of dipoles in the thermodynamic limit, as opposed to the quantum case. The classical extremal energy configurations of a magnetic dipole system (either minimum or maximum) is one of equilibrium in which no torque should act on any given dipole.

Now, the total energy of a system of dipoles in terms of (2) can also be cast in the form of the Hamiltonian

3 $$\begin{aligned} H= & {} \frac{1}{2} \,\mathcal {C}_E \,\sum _{i \ne j} \mathbf{m}_i^T \mathbf{J}_{ij}~ \mathbf{m}_j, \\\mathbf{J}_{ij}= & {} \frac{1}{\Vert \mathbf{r}_{ij}\Vert ^3} \left( \mathbf{I} - 3~\frac{ \mathbf{r}_{ij} \times \mathbf{r}_{ij} }{\Vert \mathbf{r}_{ij}\Vert ^2} \right) . \end{aligned}$$

The quadratic form (3) will be particularly suitable in the next section.

## Luttinger–Tisza and energy decomposion methods

We can obtain the spectrum of a system of identical dipoles using the Luttinger–Tisza method^17^ under the assumption that the minimum energy configuration exhibits translational symmetry. If *T*(*i*) denotes the points generated from *i* with discrete translations belonging to the *T* symmetry group the mentioned symmetry corresponds to $\mathbf{m}_i=\mathbf{m}_{i'}$ for all $i' \in T(i)$. The system can be split into identical cells and thus limit the summation to one single cell. Therefore, the energy per dipole can be expressed as

4 $$\begin{aligned} E = \frac{1}{2n}~ \sum _{i,j=1}^{n} \mathbf{m}_i^T \mathbf{A}_{ij}~ \mathbf{m}_j, \end{aligned}$$

where *n* is the number of dipoles per cell, and $\mathbf{A}_{ij}$ are (symmetric) matrices defined by

5 $$\begin{aligned} \mathbf{A}_{ij} = \sum _{ j' \in T(j),~ j' \ne i } \mathbf{J}_{ij'}. \end{aligned}$$

The energy per dipole finally reads as

6 $$\begin{aligned} E = \frac{1}{2n}~ \hat{\mathbf{m}}^{T} \hat{\mathbf{A}}~ \hat{\mathbf{m}}. \end{aligned}$$

The method involves the sotution of an eigenvalue problem of the *nd* dimensional matrix $\hat{\mathbf{A}}$ (*d* is the dimension of the dipole), with $\lambda _k$ being the eigenvalues and $\hat{\mathbf{x}}_k$ the corresponding (orthogonal) eigenvectors of the system, with $\Vert \hat{\mathbf{x}}_k\Vert =\sqrt{n}$. The final expression for the energy reads as

7 $$\begin{aligned} E = \frac{1}{2}~ \sum _{k=1}^{nd} \lambda _k a_k^2, \end{aligned}$$

where $a_k$ denotes the components of $\hat{\mathbf{m}}$ in the base $\{\hat{\mathbf{x}}_k\}$.

Two conditions must be satisfied for $i=1 \dots n$, namely

8 $$\begin{aligned} \left\| \sum _{k=1}^{nd} a_k \mathbf{x}_k^i \right\| = \mu ,\,\,\,\,\,\, \sum _{k=1}^{nd} a_k^2 = \mu ^2. \end{aligned}$$

Within the framework of the Luttinger–Tisza method, these two constraints are known as the strong and the weak conditions, respectively. We can thus obtain the minimum energy per dipole as $E_{min}=1/2~\lambda _{min}~ \mu ^2$, where $\lambda _{min}$ denotes the smallest eigenvalue of $\hat{\mathbf{A}}$.

**Figure 1:**
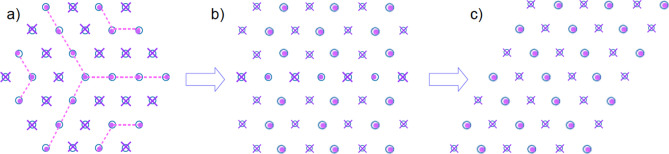
Depiction of the minimum energy configuration for perpendicular dipoles in a single layer, as we distort the lattice on which we grow the dipole crystal. From an hexagonal shape (**a**), whose ground state is the flag state with energy -0.919515, we obtain another minimum energy configuration where the shape is square-like, as shown in (**b**), with energy − 0.883517. Distorting the previous shape into a parallelogram (**c**), we basically obtain the first configuration (1/6th of it), with the same energy − 0.919515. Crosses represent dipoles pointing inwards, whereas dots correspond to dipoles coming out of the plane. See text for details.

**Figure 2:**
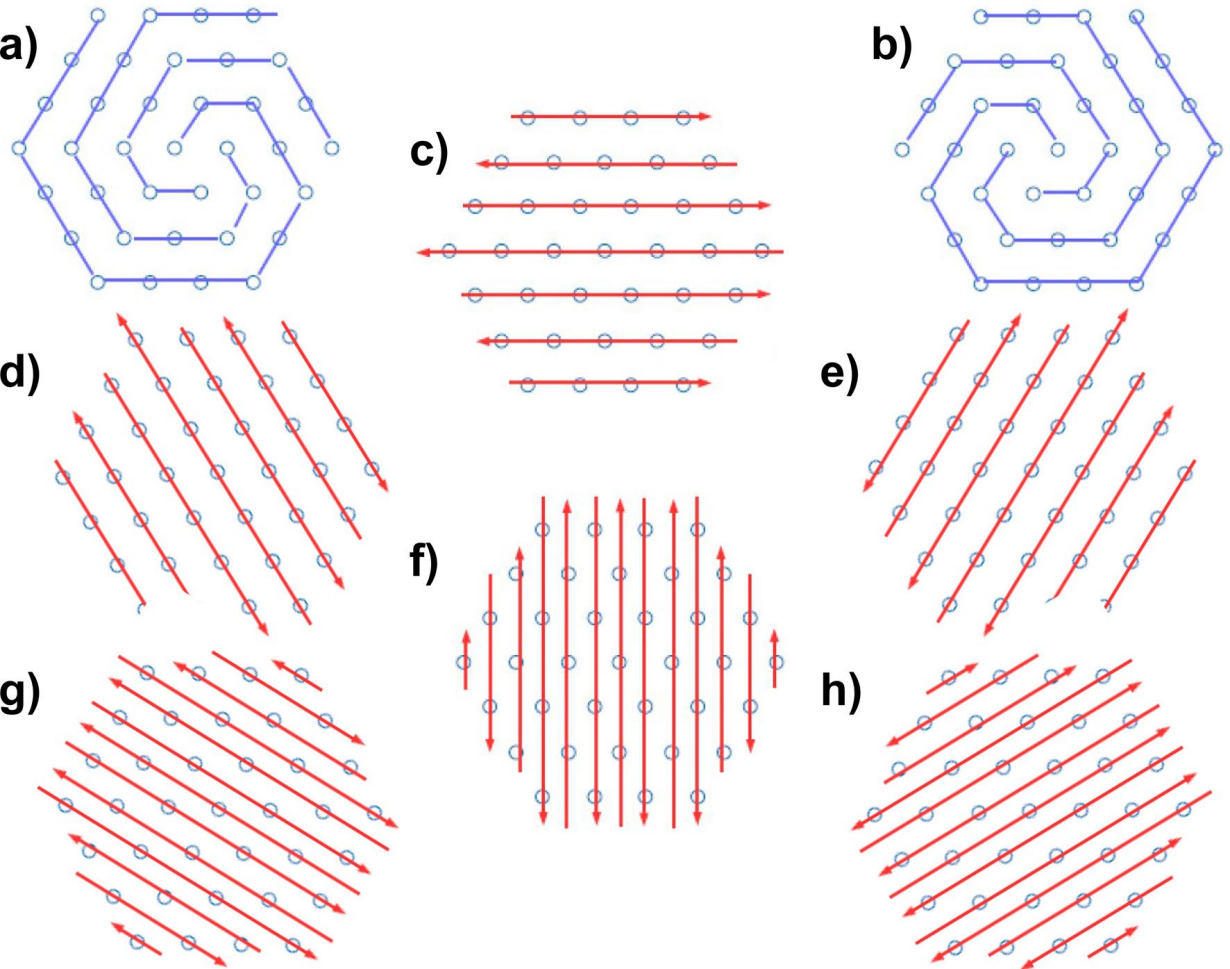
Depiction of all configurations as obtained by the Luttinger–Tisza method, or employing symmetry arguments. Configurations (**a**) and (**b**) correspond to the minimum energy, the vortex, which is − 2.758545. All dipoles in (**a**) point clockwise, and counter-clockwise in (**b**). Configurations (**c**–**e**) all (antiferro) have the same energy, − 2.04746. They are six-fold degenerate (the other three corresponding to all dipoles being flipped). Finally, configurations (**f**–**h**) (also six fold-degenerate, remaining three not shown) all possess (also antiferro) the maximum energy, namely, 2.96697. See text for details.

There exists an alternative to the previous Luttinger–Tisza method, derived by us^36–38^. For a certain set of positions $\{\mathbf {r_1}, \mathbf {r_2},\ldots , \mathbf {r_M} \}$ corresponding to *M* identical dipoles (all of them with the same magnitude $\mu$), one resorts to a minimization procedure for its total dipole–dipole interaction energy. We chose the well-known *simulated annealing*^39^ (SA) method. This Monte Carlo method is able to scape from local minima in the space of *M*(2*M*) variables corresponding to 2*D*(3*D*). We have checked that even for high values of the total dimensionality of the problem, it returns precise values for the optimal energy.

For a finite sample of *N* dipoles it is then easy to infer the minimum energy configuration. Once it is known, one needs to “grow” the plaquette in the most symmetric way, that is, equally in all possible directions. In the same spirit of the liquid drop model^40^, which considers the total nuclear energy as a sum of different contributions, such as the *volume* or the *surface* term, we shall correspond each inferred total energy $E_N$ to a series of functionally different terms depending on *N*, which fixed coefficients to be obtained later on.

Therefore, the total energy is decomposed as

9 $$\begin{aligned} E_N\,=\,N\,V_E \,+\,N^{1/2}\,S_E\,+\,N^{1/3}\,L_E\,+\,C. \end{aligned}$$

The term $\propto N$ is the *volume* contribution, in other words, the asymptotic energy per dipole in the thermodynamic limit. That is, $V_E\,\equiv \,\lim _{N\rightarrow \infty } E_N(N)/N$. The second term in (9) corresponds to the *surface* contribution. Similarly, the third term in (9) takes into account those dipoles on the boundary $\partial \Omega$. Finally there is an independent contribution. By adjusting the numerically obtained total dipole–dipole plaquette energy to the functional form (9), which employs the so-called Levenberg-Marquardt^41^ non-linear regression, we do obtain remarkable results. In this fashion, we are able to infer the value of $\lim _{N\rightarrow \infty } E_N(N)/N$ with great precision.

The previous overall *optimal configuration* − *sample growing* scheme, constitutes a easy-to-implement alternative to Luttinger’s because (1) no periodic boundary conditions are imposed, nor (2) the computation of Ewald sums (given in terms of involved lattice sums^42^) are required for obtaining the minimum energy per dipole in the limit $N \rightarrow \infty$.

Also, as a general rule in any two-dimensional periodic configuration of dipoles (not necessarily optimal ones), we have discovered that every global rotation of all dipoles by a phase $\theta$ is followed by a linear dependency of the total interaction energy on the cosine of *twice* the phase. That is, $E(\theta )\,=\,a\,+\,b\,\cos (2\theta )$. Heuristically, this relation can be obtained as a generalization of the 1D case. Suppose that all dipoles along an infinite straight line are rotated clockwise by an angle $\theta$. The total (minimum) energy per dipole in the thermodynamic limit will be given by $E(\theta )=(1-3\cos ^2\theta ) \zeta (3)$, where $\zeta$(3) is the so called Apéry constant. By using the relation between the cosine of an angle and half that angle, we obtain $E(\theta )=-1/2\,\zeta (3) -3/2\,\zeta (3)\cos (2\theta )$.

**Figure 3:**
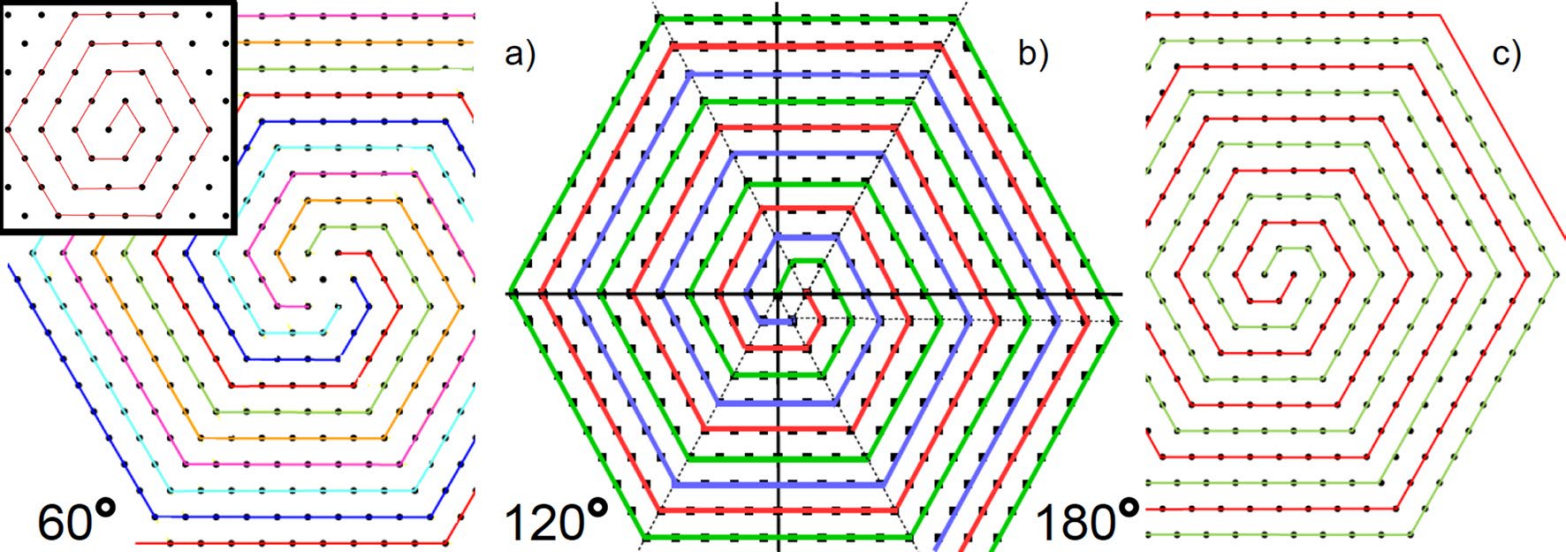
Plot of the different sets of spirals equivalent from the symmetry point of view (except the one in the inset) to the vortex state, which is (**b**). Only the central one occurs. All dipoles point clockwise. See text for details.

**Figure 4:**
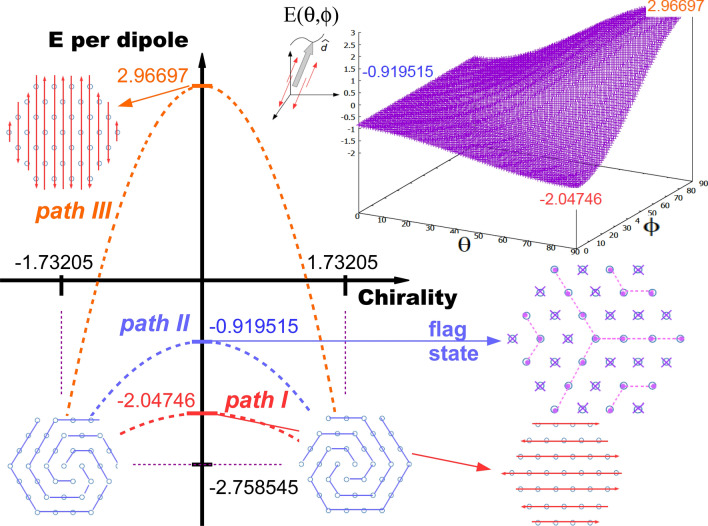
Plot of the different paths that can lead to the appearance of the vortex ground state by virtue a spontaneous symmetry breaking mechanism. The role of the order parameter is played by the chirality. All states shown except the vortex can be topologically connected by the same transformation shown next to the corresponding energy landscape. Remarkably, the energy of the flag state is exactly one-third of the energy of the vortex one. See text for details.

## Two-dimensional structures

### Hexagonal lattice: out-of-plane dipoles

Let us first consider an infinite hexagonal layer where dipoles can freely rotate out-of-plane, yet always being fixed in space. The first state that one could think of is the maximum energy state, which corresponds to all dipoles either point upwards or downwards. This instance was first considered by Danilov et al.^43^, where a direct calculation of the total energy per particle in terms of lattice sums was mentioned to be divergent, which is not. In point of fact, we do obtain using our energy decomposition method the following values in (9): $V_E\,=\,5.51501 \pm 2.87\times 10^{-5}, S_E\,=\,-49.4838 \pm 0.08977, L_E\,=\,110.935 \pm 0.6583$ and $C\,=\,-255.779 \pm 5.44$. Since we are interested in $\lim _{N\rightarrow \infty } E_N/N\,=\,5.51501$, this is the value obtained.

Interestingly, the minimum energy configuration when we grow the crystal of dipoles in hexagonal plaquettes corresponds to a special arrangement of dipoles as shown in Fig. 1. Crosses represent dipoles pointing inwards, whereas dots correspond to dipoles coming out of the plane. We call this configuration the “flag” state, due to its resemblance with the South African flag. Notice the special arrangement of the dipoles, that makes this state posses a point symmetry of $120^{\circ }$. This state is certainly unique from the topological point of view. Also, we notice that it is highly sensitive to the boundary $\partial \Omega$. The transition from the flag state Fig. 1a to a new state whose boundary is square-like Fig. 1b is also shown. This configuration possesses a higher energy, namely, $-0.883517 \pm 1.852\times 10^{-10}$. When the lattice is further distorted to form a parallelogram, we recover 1/6th of the original state in Fig. 1c. It is obvious that the shape of the boundary affects the total energy per dipole all the way to the thermodynamic limit, a feature which is not blurred by finite-size effects. We currently can only offer energetic numeric arguments in order to explain the existence of the flag state, but no physical ones. Notice that the high sensitivity of the flag state to the boundary conditions is an inherently typical feature of topological states in the quantum sense. In other words, in the quantum realm, it is very difficult to rigorously prove how the boundary $\partial \Omega$ affects the robustness of the corresponding enclosed state, and that is no different classically.

All that is known to happen is that the flag state is not topologically equivalent to the other two configurations. There is a clear symmetry reduction from going to the flag state to the others, an effect that has energetic consequences only in the square-like confined one, the one in Fig. 1b. It is likely that first and second configurations (**a**) and (**b**) in Fig. 1 tend to the last one (**c**) there in the bulk.

### Hexagonal lattice: in-plane dipoles

The Luttinger–Tisza method was applied to a system of two-dimensional dipole moments with identical scalar strength located at the sites of an infinite rhombic lattice with an arbitrary rhombicity angle by Brankov and Danchev^44^. This two-dimensional cell choice allowed to study the simple square lattice and the hexagonal one in a very compact way. Their study was an extension of two previous works by Belobrov et al.^45,46^ that considered *finite* clusters of dipoles, highlighting the existence in the hexagonal case of a “macro-vortex”. Brankov and Danchev^44^ extended their original work and provided a better numerical accuracy to the computation of configuration energies. Although further work on dipole–dipole interacting systems followed^47–55^ inspired by the Luttinger and Tisza seminal work^17^, Refs.^44–46^ constitute the first consistent treatment of simple square lattice of dipoles and, specifically, the hexagonal lattice one. We shall provide an extended description of the hexagonal case in the present work, with an increased precision of the energy per dipole in the thermodynamic limit by several orders of magnitude.

Figure 2 depicts the set of possible states obtained by either employing the Luttinger–Tisza method, or using numerical computations based on symmetry arguments. Each configuration is shown, along with their energy per dipole energies, given with exact decimal digits. Incidentally, the antiferromagnetic states of energies − 2.04746 and 2.96697 are connected by a global rotation in the sense of the $E(\theta )\,=\,a\,+\,b\,\cos (2\theta )$.

Let us consider the case of the minimum possible energy configuration given by the vortex. The Luttinger–Tisza method provides an energy given by the lattice sum

10 $$\begin{aligned} E_0\,=\,-\frac{1}{2} \sum \limits _{r\in L^2,r\ne 0} \frac{1}{r^3}, \end{aligned}$$

where $L^2$ stands for the points in the hexagonal lattice. There are means to speed-up the computation of lattice sums such as (10) by using Ewald sums.

What we would like to stress out is that we shall compute the very same value for (10) without the burden of even considering the Luttinger–Tisza method at all, with all the corresponding formalism. We shall, then, show how our energy decomposition method works. Incidentally, we consider a slight deviation of (9) of the form $$N\,V_E \,+\,N^{1/2}\,S_E\,+\,C$$, where the boundary term is not accounted. Recall that we are only interested in the bulk value $$V_E $$, and this omission shall not imply any variation in the final result whatsoever. Once we have obtained several hexagonal clusters of increasing size *N* their corresponding energies (according to Fig. 2a or 2b), the concomitant non-linear fit returns the values $$V_E = -2.758545\pm 8.223\cdot 10^{-9}, S = 1.22402\pm 7.662\times 10^{-6}, C= 3.30666\pm 0.001497$$. Extraordinary precise values for $$E_0$$ (10) as well as for the other energies are thus obtained in the same fashion without employing the tools required for the Luttinger–Tisza method.

Let us focus on the description of the vortex state. First of all, it is not expected to have a peculiar configuration of in-plane dipoles as the minimum energy state, having a considerable energy of − 2.758545. As opposed to the flag state, the vortex state is very much robust and, as far as our numerical computations are concerned, does not depend on the boundary $\partial \Omega$ of the system. The vortex state is depicted in Fig. 3b in great detail. Let us analyze the vortex state from the symmetry point of view. The union of three spirals (red, green and blue) has a third-order axis (rotation on 120$^{\circ }$) perpendicular to the plane of the lattice. This is a point group of symmetry 3. The symmetry group of the hexagonal lattice contains identical transformation, 2-, 3- and 6th order rotations and six reflections. The identical transformation leads to one spiral, shown in the inset of Fig. 3, and rotations lead to the spiral configuration that forms the vortex state.

What is remarkable about the vortex state from the symmetry perspective alone, is that the partition of the hexagonal lattice into spirals is not unique. One can construct the partition on two spirals with the second-order axis (the point group of symmetry 2, rotation of 180$^{\circ }$ as in Fig. 3b. Furthermore, one can also obtain the partition of the hexagonal lattice on the central point with 6 spirals with the six-order axis (rotation on 60$^{\circ }$, the point group 6), which constitutes the vortex in Fig. 3a. Of course, all these groups are subgroups of the symmetry group of the hexagonal lattice.

From the symmetry point of view, all spirals in Fig. 3 (except the one in the inset) are equivalent. In point of fact, the energy per dipole in the bulk is the same for the three cases. Thus, we are left with the question of why the only one that occurs is the vortex state with $120^{\circ }$-symmetry.

#### The vortex structure and the onset of spontaneous symmetry breaking

Despite the previous description, the question still remains: why a (macro)vortex? The works^45,46^ managed to answer the question for *finite* clusters of dipoles, based on surface magnetic charge arguments. However, when dealing with the corresponding $N\rightarrow \infty$, a more intuitive reason arises: “The structure of a dipole system should be sensitive to the symmetry of the nearest-neighbor environment”. However, this claim is rather vague.

One possible explanation could be the following. Let us assume that the minimum energy configuration for low temperatures is the (in-plane) ferromagnetic one in the bulk. The original Hamiltonian (3) has an *O*(2) dipole rotation symmetry, whilst the direction of the ferromagnetic order is not fixed for $N\rightarrow \infty$. Things, however, change near the edges in *finite* systems. There there is a clear ferromagnetic direction, and different regions or domains merge from the boundary to the interior, in a sort of inwards dipole “freezing”.

The previous argument somehow implicitly assumes the six-fold symmetry of the vortex state as inferred from the lattice. There is no reason why ferromagnetic domains should not merge forming a triangular shape, for instance. Again, it is not quite convincing. In essence, the existence of vortices formed by magnetic dipoles have been suggested mainly by numerical simulations. Analytic Taylor expansion of the dipole–dipole interaction has shown^56^ that it is able to obtain a local representation in the plane such that the lattice symmetry induces a magnetic anisotropy. This approximation, although purely mathematical in essence, is somehow capable of predicting the existence of vortices in the ground state magnetization.

Based on the previous facts, we shall pursue an explanation solely based on physical and symmetry arguments. To such an end, we shall first define the *chirality* of a set of *N* dipoles $\{\mathbf{m_i}\}$ with respect to an arbitrary, perpendicular axis *o* as

11 $$\begin{aligned} \xi _N\,\equiv \,\left\| \sum \limits _{i=1}^N \mathbf{r_{i}}\,\times \, \mathbf{m_i} \right\| , \end{aligned}$$

where $\mathbf{r_{i}}$ denotes the position of the dipole $\mathbf{m_i}$ with respect to an axis *o*. The chirality is obviously a quantity independent of the position of the axis. In the case of the vortex state, we obtain the following result

12 $$\begin{aligned} \xi _N\,=\,V_1\,N^3\,+\,V_2\,N^2\,+\,V_3\,N, \end{aligned}$$

with $V_1=1.73205\pm 1.833\times 10^{-7}$, $V_2=7.79359\pm 2.491\times 10^{-4}$, and $V_3=11.4368\pm 0.08078$. Therefore, we shall define the chirality of the system of dipoles in the thermodynamic limit as $\xi \,\equiv \,\lim \limits _{N\rightarrow \infty } \xi _N/N^3$, which is equal to $\pm 1.73205$, depending on the vortex pointing clockwise or counter-clockwise.

The magnetization of the vortex state is per se rather intriguing. The three embedded spirals conform an overall state with a nonzero total dipole moment *M*. Since the number of dipoles *N* goes as $1+3n(n-1)$ (formula for hexagonal centered numbers) for each added layer *n*, and *M* is easily found to be $(2n-1)\mu$, we thus have the explicit dependence

13 $$\begin{aligned} M\,=\,\frac{\mu }{3}\,\sqrt{12N\,-\,3}. \end{aligned}$$

Therefore the magnetization per dipole of this state goes as $1/\sqrt{N}$, as opposed to the expected 1/*N* behavior in the thermodynamic limit. Notice that in the rest of states depicted in Fig. 2, *M* is either 0 or *o*(1/*N*). The magnetization per dipole could be a potential physical magnitude to be used in order to link the minimum energy configuration of the hexagonal lattice, that is, the vortex, with the rest of the spectrum. However, it does not change continuously and remains practically zero for all practical purposes. That is why we shall consider the aforementioned chirality $\xi$. In the language of phase transitions, this is going to be our *order parameter* in the Landau sense, although at zero temperature.

In order to explain the appearance of a vortex structure as the minimum energy configuration of interacting dipoles in the hexagonal lattice we shall invoke here a mechanism of **spontaneous symmetry breaking**. Phases of matter, such as crystals, magnets, and conventional superconductors, as well as simple phase transitions can be described by spontaneous symmetry breaking (See^58,59^ and references therein). In the case of crystals as periodic arrays of dipoles, for instance, they are not invariant under all translations (only under a small subset of translations by a lattice vector). The Hamiltonian of our system (3), for in-plane dipoles, has a finite rotational symmetry defined by the symmetry group of the hexagonal lattice plus $Z_2$, as well as all the corresponding states depicted in Fig. 2, a symmetry that is further reduced for the ground states, the vortex states. The vortex clearly breaks this rotational symmetry. For out-of-plane dipoles, the Hamiltonian is invariant under *O*(2) rotations around any perpendicular axis. Although the flag state has $120^{\circ }$ degrees symmetry, it is indeed invariant under *O*(2), as well as the other out-of-plane states. Plus, the vortex configuration possesses a $Z_2$ symmetry, that connects configurations with opposite chiralities. Notice that $Z_2$ commutes with the Hamiltonian (3). Numerically, the vortex structure is rather robust, and already occurs for finite instances. It is extremely difficult to prove that the vortex state does not depend on the boundary conditions as we go to the thermodynamic limit, as opposed to the flag state. As previously mentioned, they easily appear in all sort of simulations. This fact does not prove anything by itself. We can only rely on numerical calculations to assert the robustness of the vortex state (recall that this procedure is also the case in the quantum realm).

All the previous states, either out-of- or in-plane dipole configurations, has exactly zero chirality *except* the vortex structure. Thus, we can consider different routes to the spontaneous symmetry breaking that lead to the formation of vortex states. They are shown in detail in Fig. 4. Path III connects the maximum and minimum energies, by a continuously development of the chirality. The same situation happens for path I, linking two states energetically close in relative terms. Path II is rather special for it brings together the flag state and the vortex state, the latter being already a special state. Remarkably, the energy of the vortex state is exactly three times that of the flag state.

All states except the vortex one can be topologically connected by the transformation shown in the upper figure, where all dipoles follow the direction of vector $\mathbf{d}$ in spherical coordinates. The corresponding energy landscape is also shown. Notice how the two configurations belonging to the in-plane hexagonal case reduce to 1/6th of the flag state when $\theta \rightarrow 0$.

The spontaneous symmetry breaking mechanism that we propose here use the chirality as the order parameter, which induces the appearance of a special configuration of dipoles having the minimum possible energy. It does not necessarily imply that the shape has to be that of a (macro)vortex, but at least a different one from the set of possible states possessing a clear, differentiated symmetry.

### The honeycomb lattice: topology of the ground state

**Figure 5:**
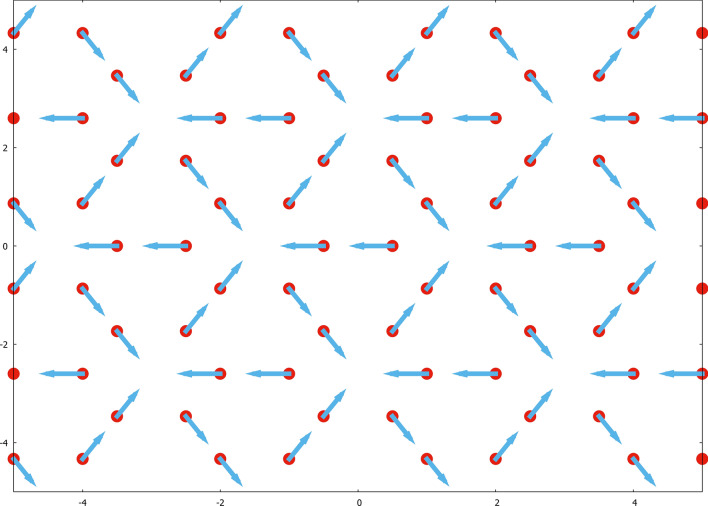
The honeycomb lattice and the corresponding dipole configuration of minimum energy. Notice that every dipole sink coordinates with six local vortices. See text for details.

In the honeycomb lattice, and following our procedure, a sufficiently big sample of dipoles unveils the disposition of the dipoles for the minimum energy configuration. This structure is shown in Fig. 5, along with the lattice. The minimum energy per dipole in the thermodynamic limit is found to be $-2.22691 \,\pm \,6.322\times 10^{-6}$, higher than the one corresponding to the hexagonal lattice.

We do not observe a single dipole structure, as in the case of the vortex in the hexagonal lattice, with ferromagnetic domains. Antiferromagnetism is not present either. Instead, the system is formed by an array of local sinks and vortices. For every sink, we have six local vortices that surrounds it. As in all classical ground states with no geometric frustration^57^, it is not highly degenerate (only two) and the total magnetization is zero.

Certainly, we cannot possibly have the topology present in skyrmion/bimeron spin textures, yet we can obtain a periodic arrangement of interesting structures such as sinks and vortices.

## Three-dimensional structures

### Hexagonal simple lattice

The problem of obtaining the minimum energy per particle in the bulk of a system of interacting dipoles is interesting per se, regardless of the peculiarities of the corresponding dipole topology. Also, to the best of our recollection, it has not been studied previously in the literature.

Again, in order to tackle the problem, we use our methodology. After placing $N_z\in [1,100]$ layers of hexagonal lattices in parallel, each one of which with side $N_x=N_y=20$, we conclude that no dipole projection onto the x–y plane occurs. Of course, there are finite-size effects that account for dipoles than can be found not being perfectly perpendicular, but these effects are blurred in the thermodynamic limit. In point of fact, if the 2D layer retains its hexagonal shape, the final state of the entire column corresponds to layers of flag states one on top of the other. On the contrary, if the 2D layers possess some other shape, such as a parallelogram, the crystal will look like a 1/6-prism of the latter. As in the 2D case, we know that the two vertical arrangements will possess the same energy, the same zero magnetization, but the flag hexagonal lattice dipole crystal will display a particular topology, not present in the 1/6-prism. In both cases, either flag layers or a 1/6-prism, we shall have multi-ferromagnetic-domain structures of dipoles pointing up or down along the *z*-axis.

**Figure 6:**
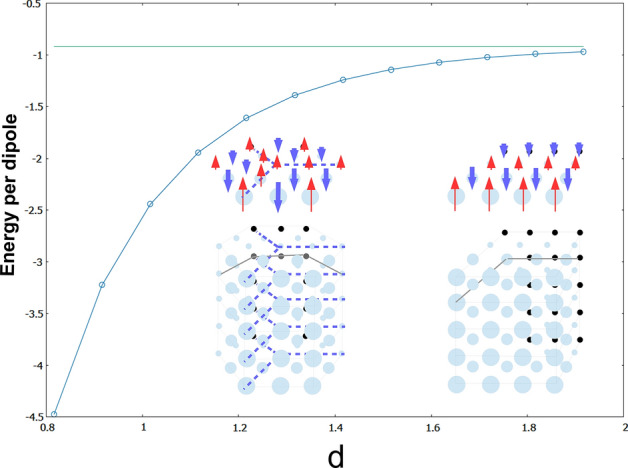
Plot of the evolution of the energy per particle in the thermodynamic limit as a function of the vertical cell distance *d*, for both the flag-state crystal and the 1/6-prism. The horizontal line corresponds to the asymptotic $d\rightarrow \infty$ energy per particle value of the flag state. See text for details.

**Figure 7:**
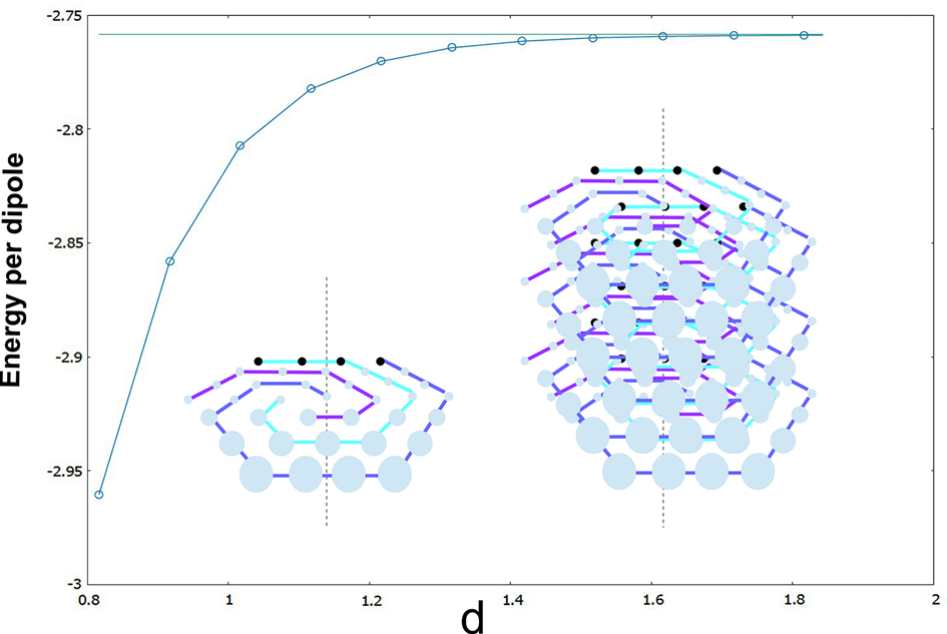
Plot of the evolution of the energy per particle in the thermodynamic limit as a function of the vertical cell distance *d*, for the hcp lattice. The horizontal line corresponds to the asymptotic $d\rightarrow \infty$ energy per particle value of the vortex state. See text for details.

This is the case for any *d* value in the rhombic cell. Thus, *d* shall correspond to the interlayer distance, measured in units of the hexagonal lattice constant *a*. Of course, there is no preferred value *d*. Therefore, it is mandatory to perform an exhaustive numerical exploration in order to obtain the minimum energy per dipole in the bulk and for every different *d* value. The results of the computations are depicted in Fig. 6. The small spheres in Fig. 6 and 7 denote the position of each dipole in the crystal, which could easily be regarded as the position of the nuclear magnetic moment corresponding to the nucleus of a certain species. The representation using spheres also helps to better understand the later comparison between different three-dimensional structures in terms of hard spheres.

The total interaction energy monotonically decreases as *d* decreases, which implies that the system is bounded. The first computed point is for $d=\sqrt{2/3}a$, which corresponds (for later comparison) to the minimum interlayer distance in the case of the hexagonal closed packed lattice, for $a=b=1$. As the interlayer separation increases, the interaction between layers becomes negligible, and we recover asymptotically the expected value for one single layer, namely, − 0.919515. Here we list the computed values for several distances *d*.

**Table:** 

d	$E_{\min }$
$\sqrt{\frac{2}{3}}\,=\,$0.816496581	− 4.47554
$\sqrt{\frac{2}{3}}+0.1\,=\,$0.916496581	− 3.22521
$\sqrt{\frac{2}{3}}+0.2\,=\,$1.01649658	− 2.44428
$\sqrt{\frac{2}{3}}+0.3\,=\,$1.11649658	− 1.94183
$\sqrt{\frac{2}{3}}+0.4\,=\,$1.21649658	− 1.61168
$\sqrt{\frac{2}{3}}+0.5\,=\,$1.31649658	− 1.39146
$\sqrt{\frac{2}{3}}+0.6\,=\,$1.41649658	− 1.24296
$\sqrt{\frac{2}{3}}+0.7\,=\,$1.51649658	− 1.14203
$\sqrt{\frac{2}{3}}+0.8\,=\,$1.61649658	− 1.07302
$\sqrt{\frac{2}{3}}+0.9\,=\,$1.71649658	− 1.02563
$\sqrt{\frac{2}{3}}+1.0\,=\,$1.81649658	− 0.992991
$\sqrt{\frac{2}{3}}+1.1\,=\,$1.91649658	− 0.970449

All values for the energy are given with exact decimal digits. The most reasonable value is around $d=1$, for the $\sqrt{\frac{2}{3}}$-one is relatively too close in the case of the simple hexagonal 3D lattice. Of course, one could have also tackled the problem by employing the Luttinger–Tisza method, but as explained earlier, our method is much faster, and avoids computing typically involved lattice sums.

The present case shows that the results obtained for dipoles pointing off-plane, either in the form of flag states of alternating rows of dipoles pointing up and down, are exactly reproduced in the extension to 3D where the layers are simply piled up, forming the simple hexagonal lattice. In this case, the dipole crystal displays a null chirality as well.

### Hexagonal closed packed lattice

The problem of finding the minimum energy configuration of identical dipoles interacting in the hexagonal closed packed lattice (hcp) is arguably the most difficult case considered. By employing a similar numerical approach as in the case of the simple hexagonal lattice in 3D, the first aspect which is readily found is that all dipoles lie on the x–y axis. Again, and due to the optimization of finite samples, finite-size effects appear, where dipoles on the boundaries may have spurious vertical projections. However, in the thermodynamic limit, there is no vertical component for the magnetization. Furthermore, and this is what is more remarkable, all layers are formed by vortex states, which provide a rather intricate image of how dipoles are arranged inside the crystal. The corresponding image is depicted in the inset of Fig. 7.

Once the optimal configuration is found, we shall use our energy decomposition method to find the energy per dipole in the bulk. Since different values for the *d*-value are possible, we provide several ones starting from the closest one $d=\sqrt{2/3}$ (hard spheres touching). The corresponding values are listed in the following:

**Table:** 

d	$E_{\min }$
$\sqrt{\frac{2}{3}}\,=\,$0.816496581	− 2.96042
$\sqrt{\frac{2}{3}}+0.1\,=\,$0.916496581	− 2.85824
$\sqrt{\frac{2}{3}}+0.2\,=\,$1.01649658	− 2.80741
$\sqrt{\frac{2}{3}}+0.3\,=\,$1.11649658	− 2.78242
$\sqrt{\frac{2}{3}}+0.4\,=\,$1.21649658	− 2.77025
$\sqrt{\frac{2}{3}}+0.5\,=\,$1.31649658	− 2.7643
$\sqrt{\frac{2}{3}}+0.6\,=\,$1.41649658	− 2.7614
$\sqrt{\frac{2}{3}}+0.7\,=\,$1.51649658	− 2.75999
$\sqrt{\frac{2}{3}}+0.8\,=\,$1.61649658	− 2.75931
$\sqrt{\frac{2}{3}}+0.9\,=\,$1.71649658	− 2.75897
$\sqrt{\frac{2}{3}}+1.0\,=\,$1.81649658	− 2.75881
$\sqrt{\frac{2}{3}}+1.1\,=\,$1.91649658	− 2.75872

Again, the energy values are given with exact decimal digits. As expected, when the interlayer separation is high enough, the energy per particle reduces to the value in the vortex state, that is − 2.758545.

In the case of hexagonal closed packed structures, dipoles follow in each layer the configuration of minimum possible energy for in-plane dipoles, displaying defined regions of ferromagnetic domains. The chirality, as opposed to the previous hexagonal lattice, is clearly present here. Also, as opposed to the hexagonal lattice, there is a unique minimum configuration possible which, as opposed to the flag state, is not altered by the shape of the crystal sample. For the same *d*-value (greater or equal to *a*), the hcp lattice is energetically more bounded, a feature which is somehow intuitively expected.

Our results for the hcp lattice, with vortex layers, are not to be confused with helical spin configurations in hexagonal crystals^60^. On the one hand, the former occur in simple hexagonal lattices, and on the other, their explanation is due to the a positive interaction between nearest neighbors and a negative interaction with second-nearest neighbors. Therefore, rotations in planar layers occur only if spins are treated classically as dipoles in the Heisenberg exchange Hamiltonian $-|J_1|\,\cos \alpha + |J_2|\,\cos (2\alpha )$, where $\alpha$ is the angle of rotation of the dipoles in the layers forming an helix.

## Discussion

We have described the emergence of the robust vortex state as the minimum energy configuration possible mediated by a mechanism of spontaneous symmetry breaking. In a way, since $T=0$, we are describing a classic topological transition driven by the continuous chirality $\xi$ parameter.

The real arena where the symmetry breaking should be considered is at finite temperature, and not to employ the internal energy *E* or *U*, but the (Helmholtz) free energy $F = E - T\cdot S$ instead (a collection of particles will always seek to minimize its free energy). There, one should take into account that the entropy *S* clearly scales differently as opposed to the total energy *E*. Scaling here is referred to the size of the system. In other words, depending on the density of states of the system, the entropy may have a scaling with system size different than the internal energy. Therefore, the ensuing study should become more involved.

However, all the present contribution is described at zero temperature. It the temperature was finite, as it was lowered, the entropy would provide a decreasing contribution to the free energy, and the system would at some point fall into another state. This usual temperature-driven analysis has not been implemented here, for again we are treating the system at zero temperature. However, the Landau approach for finite *T* may not to be much different from what has been presented here. We are confident that, in a finite temperature analysis, our conclusions about the feasibility of the spontaneous symmetry breaking explanation for the existence of vortex would still hold.

Energies and configurations have been computed either by recourse to the Luttinger–Tisza method, or by employing our energy decomposition approach, which has been proved successful in other scenarios involving the computation of energies based on the dipole–dipole energy interaction of periodic systems in the thermodynamic limit.

The topology of the equilibrium configurations for out-of-plane dipoles has provided a new state, the flag state. We have noticed that it is very sensitive to the boundary conditions (as opposed to the vortex case), much like topological phases in the quantum case. Besides the usual states in the in-plane case for the hexagonal lattices, we have computed the exact minimum energies per dipole for the three-dimensional cases as a function of the interlayer distance. Remarkably, in the simple hexagonal case the layers are formed by flag states, whereas in the hexagonal closed packed configuration, the layers are the vortices themselves. Also, an energy analysis shows that the hexagonal closed packed system is more bound.

Summing up, we have presented that, within the limits of the classic dipole–dipole interaction, one can observe non-trivial topological structures that have special interest, in particular the minimum energy vortex configuration. Thus, we showed that particular configurations of dipoles are not exclusive to quantum spin textures such as skyrmions or bimerons, but also occur in the special case of triangular or hexagonal lattices.

## Data Availability

The data are available upon reasonable request at jbv276@uib.es.
